# Assessing pain and analgesic consumption after the use of Kinesio tape in web strip technique comparing with cryotherapy after impacted third molar extraction: a randomized clinical split-mouth study

**DOI:** 10.1038/s41405-025-00296-x

**Published:** 2025-02-12

**Authors:** Belal Alhourani, Mazen Zenati, Ahmad Alnada

**Affiliations:** 1 Department of Oral and Maxillofacial Surgery, Faculty of Dentistry, Damascus University, Damascus, Syria; 2 Department of Periodontology, Faculty of Dentistry, Damascus University, Damascus, Syria

**Keywords:** Maxillofacial surgery, Third molar removal

## Abstract

**Background and objectives:**

There are various methods to improve the patient’s quality of life and alleviate complications after surgical extraction of the lower third molar. The use of Kinesiotape is one of the most important methods used recently. This study aimed to assess the effectiveness of Kinesiotape in alleviating complications after surgical extraction of the lower third molar compared to cryotherapy.

**Methods:**

The study was designed as a randomized controlled clinical study according to the split-mouth technique and included 25 patients who had radially symmetrical lower third molars. The patients were divided randomly into two groups, and Kinesiotape (KT) using the Web-strip technique was applied in the study group (KT), while cold packs were applied in the control group Non-Kinesiotape (N-KT). The studied variables were pain, and analgesic pills consumption. These measurements were evaluated in the first five days after surgery using the visual analog scale (VAS) and the number of consumed analgesic pills.

**Results:**

The pain index value in the KT group was (60.4 ± 19.8) on the first day, and (60.4 ± 17.8) in the N-KT group. The number of analgesic pills consumed in the KT group was (3.3 ± 1.3) on the first day, and (3.4 ± 1.3) in the N-KT group. No significant statistical difference was found in pain scores according to the VAS index and the number of consumed analgesic pills between the two groups.

**Conclusion:**

The current study concluded that pain and analgesic pills consumed were similar between the two study groups, without one being superior to the other.

## Introduction

One of the most frequently performed surgical procedures in oral and maxillofacial surgery is the surgical extraction of the third molar. This extraction is often accompanied with many complications, such as pain, swelling, and trismus, which vary in severity and impact on the patient’s quality of life [1]. Therefore, various pharmaceutical and non-pharmacological methods have been used to control and alleviate these complications [2].

These methods include non-steroidal anti-inflammatory drugs, corticosteroids, proteolytic enzymes, low-level lasers, surgical drainage tube, compression bandage, and platelet-rich fibrin [3].

The use of cold packs is considered the most commonly used method of cryotherapy. Cryotherapy is one of the most commonly used methods as it is simple and inexpensive. Therefore, most studies recommend applying cold packs for 10–20 min on and 10–20 min off during the first 12–24 h after surgery [4, 5]. The topical application of cryotherapy causes changes in blood flow and constriction [6].

Cold packs applied to the treated area reduce vascular flow, restrict bacterial growth, and reduce the release of inflammatory mediators, which in turn affects edema and pain clinically. Cooling also has a local anesthetic effect in the treated area. Its effect varies depending on several factors, including the temperature, which quickly rises with application, and the amount of fat present in the skin. However, its effect is clinically proven [5, 6].

The novel studies have indicated that the use of Kinesiotape (KT) may have a significant effect on reducing pain, swelling, and trismus after surgery. KT is an elastic tape that can be stretched to 40–60% of its original length, it contains an acrylic adhesive strip in a wave pattern. KT consists of 100% high-grade cotton and elastic fibers and may include polyester in the types that are applied to sensitive skin when there is a need for higher tension to obtain the desired result. The thickness, weight, and stretchability of tapes are similar to the characteristics of the skin [7].

The tapes are water-resistant and air-permeable, and strong enough to stay for 4–5 days. In the basic product, the tape is applied to an insulating sheet at a tension rate 10–15% of its original length and contains a heat-activated acrylic adhesive. This amount of basic tension (paper off) can be used with some KT adhesive techniques. There are some clinical studies on the use of KT in the management of shoulder injuries, chronic back pain, patellofemoral pain syndrome, and lymphedema [8].

The positive effects of KT on pain and range of motion have emerged, especially when applied in the early stages of the injury. KT’s mechanism of action is based on supporting the body’s natural self-healing processes [9].

The tape returns to its original length after it is applied to the skin, creating a pulling force on the skin that leads to convolutions on the skin. This causes an increase in the interstitial distance between the skin and the connective tissue and improves the flow of blood and lymph. This leads to relieving pressure on pain receptors at the site of KT application, thus reducing the pain [10]. The effectiveness of KT in maxillofacial surgery has been tested through many surgical procedures, such as orthognathic surgery, operations on the mandibular bone and zygomatic orbital complex fractures, and surgical extraction of third molars. The application of KT using the Web Strip technique after surgical extraction of the lower third molars had a positive effect in reducing postoperative morbidity [10].

This study aims to evaluate the effectiveness of KT using the Web Strip technique in alleviating sequelae and its impact on the patient’s quality of life after surgical extraction of the lower third molars in comparison with the application of cold packs.

## Materials and methods

### Study design

**Fig. 1:**
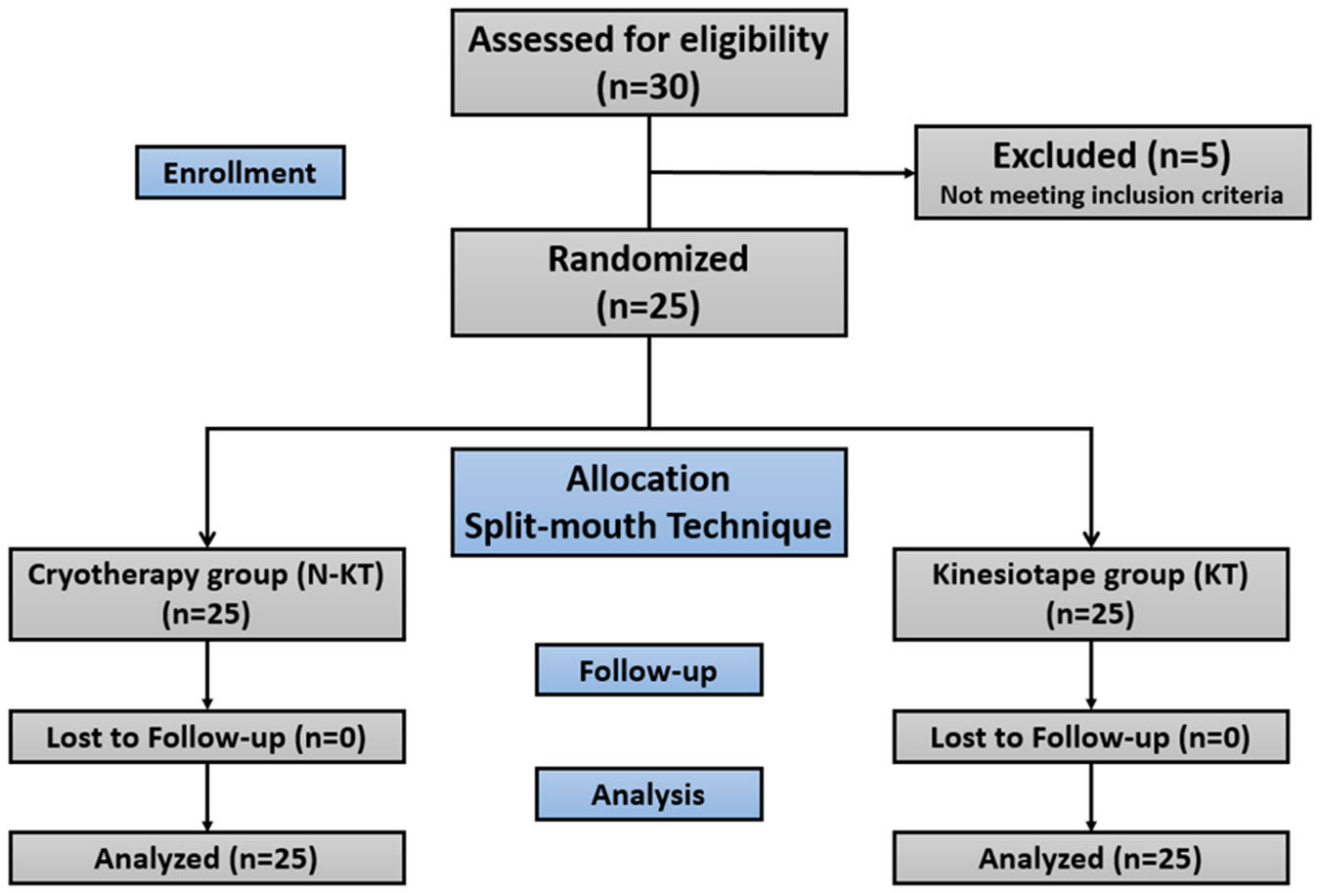
The data flow chart of the study’s patients.

The study was designed as a randomized controlled clinical study using the split-mouth technique, the study included 25 patients (Fig. 1) with radially symmetrical lower third molars from the Department of Oral and Maxillofacial Surgery, Faculty of Dentistry, Damascus University. All patients provided written informed consent according to the Declaration of Helsinki before being included in the study sample. This trial is registered in Damascus University database with ID: 1550 and in the ISRCTN with ID: ISRCTN97083158. This RCT has been written according to the new CONSORT statement. The study was approved ethically by The Biomedical Research Ethics Committee (BMREC) with the ID (DN-290424-230).

The sample was calculated based on the data of a pilot study contained five patient and used G*power software where *α* was (0.05), power (0.95) and the effect size was (1.1501) and the results were 18 sample in each group and we raised up to 25 in account of dropout of patients.

### Study sample selection

The inclusion criteria in the research sample included bilateral symmetrical lower third molars according to the angulation of impaction and moderate difficulty in extraction according to the modified Pederson scale. The ages of the patients ranged between 18 and 35 years, the duration of the surgical procedure was 20–40 min, the patients had good oral hygiene, and no temporomandibular joint disorders, and no symptoms of pericoronitis or severe pain before surgery. While the exclusion criteria were patients who are allergic to the adhesive tape material and the medications used in the research, patients who have thick hair where the material is applied and do not wish to shave it, the duration of the surgical procedure is less than 20 or more than 40 min, smoking patients, uncontrolled systemic diseases, pregnant and lactating women, poor oral hygiene, patients with temporomandibular joint disorders.

The symmetry of the angulation was determined according to the Gregory and Bell scale and according to Pederson with difficulty of extraction. Cases of moderate difficulty were selected that could be extracted under regional anesthesia in the clinic and in which the operating time and the resulting trauma often lead to measurable complications.

The study sample (50 lower third molars in 25 patients) was distributed randomly by flipping a coin to choose the side where the extraction would begin, and after the extraction to choose the side where (KT) Kinesio Tape would be applied. The time interval in performing the surgical procedure ranged between the two sides is 4 weeks.

### Surgical procedures

All surgical extractions were performed by a single surgeon under sterile conditions according to the standard surgical protocol. A mucoperiosteal triangular flap was elevated after regional anesthesia in the working area of the inferior alveolar and lingual nerves and local anesthesia of the buccal nerve using lidocaine 2% with adrenaline 1/100,000.

Then, the bone covering the molar crown began to be removed using a rounded bur, and the tooth was separated when necessary, using a cylinder bur with continuous irrigation using serum. The tooth was wiggled and extracted using elevators, and then the flap was sutured with silk thread 3/0. All patients were given post-operative instructions, but cold packs were not applied in the study group, the second surgery was performed after 4 weeks.

All patients were prescribed Augmentin 1 g (amoxicillin 875 mg + clavulanic acid 125 mg) twice a day for 7 days and paracetamol (500 mg when necessary, the daily dose must not exceed 3 g).

#### KT application

**Fig. 2:**
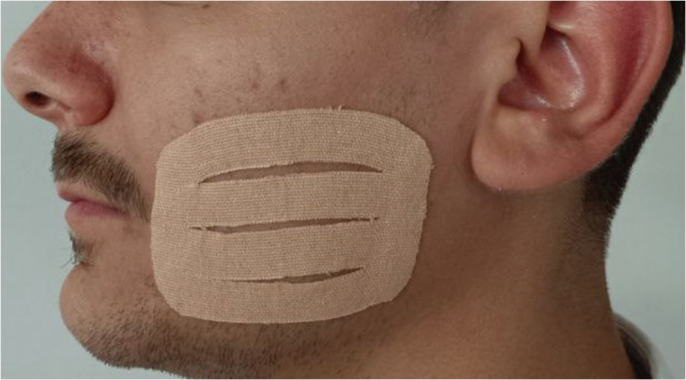
The application of KT.

Kinesio tape using the Web Strip technique was applied immediately after completing the surgical procedure and suturing, after cleansing the skin and drying it of any remaining moisture. The tape was cut to appropriate the recipient site as it is applied on the masseter area where most of the swelling occurs and where measurements are made between anatomical reference points. The shape of the applied strip is modified according to the “Web Strip” technique so that it has four elongations separated from each other in the center and connected at the two ends of the strip, in the same manner, followed in [11]. The sharp angles of the tape were rounded during cutting. The tape prepared using the Web Strip technique was adhered to the skin at the desired site using the basic tensile force applied to the tape with the insulating paper underneath (Paper Off), which constitutes 10–15% of the permissible tensile field of the tape without any excessive force (Fig. 2). The tape is rubbed from the outside after applying it to the skin to activate the acrylic adhesive. The tape was placed on the skin for 5 days without any change. The patient was warned not to manipulate the tape or expose it to excessive water or moisture. The tape used for all the study group cases was Kinesio Tex Gold Finger Print, 5 cm × 5 m (Kinesio Holding Corporation, NM, USA) with a leather-like color (beige).

#### Cryotherapy application

**Fig. 3:**
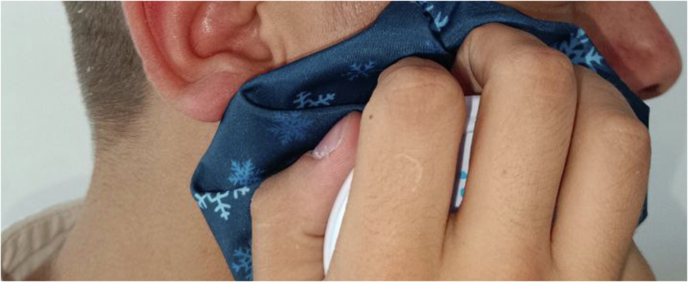
The application of cryotherapy.

Cold packs were applied to the skin of the surgical site immediately after surgery during the first day for a period of (20 min application/20 min rest) [5] on the side to which KT was not applied. The used packs were in the form of ice cubes in a cloth bag (Fig. 3).

### Study measurements

The values of the variables were recorded and collected by an independent, un-blinded external observer.

The severity of pain was studied from the day of surgery (the first day) until the fifth day after surgery by the visual analog scale (VAS), which is a numbered scale from 0 to 100, where 0 (no pain) and 100 (unbearable severe pain) [12], in addition to recording the number of consumed analgesic pills daily.

### Statistical analysis

The side to which Kinesiotape was applied was selected randomly by flipping a coin and then the data was compiled by transcribing a chart self-reported by the patient. The data were distributed normally after using Kolmogorov–Smirnov test to determine the distribution of the study data. Paired Sample T-Test was used to determine the differences between study groups. Data were collected and analyzed using SPSS V.26 (IBM, USA). The *P*-value indicating a statistically significant difference in this study was (0.05) and the power value was (0.95). The statistician was also blinded to the results of the study groups.

### Study hypothesis

#### Null hypothesis

There is no statistically significant difference in the level of pain and analgesic consumption between the application of Kinesiotape and cryotherapy when applied after extraction of impacted third molars.

#### Alternative hypothesis

There is a statistically significant difference in the level of pain and analgesic consumption between the application of Kinesiotape and cryotherapy when applied after extraction of impacted third molars.

## Results

The study sample consisted of 25 patients with each patient having two surgical sites (50 surgical sites). The mean age of the patients was (21.52 ± 1.23), and they were 10 males and 15 females (Table 1).

**Table 1:** The demographic description of study sample.

Count	Age (yr)	Gender (Male)	Gender (Female)
25	21.52 ± 1.23	10	15

### Pain index (VAS)

Pain scores according to the VAS were similar between the two groups, as there were no significant differences between the study group (KT) and the control group (N-KT) in the first five days after surgery (*P* > 0.05) (Table 2).

**Table 2:** The Descriptive and analytical statistics of pain index (VAS).

Time of measure	Control Group (N-KT)		Study Group (KT)		T-test
	Count	Mean ± SD	Count	Mean ± SD	*P*-value
1st Day	25	60.4 ± 17.8	25	60.4 ± 19.8	1.000
2nd Day	25	46.4 ± 16.5	25	46.8 ± 19.3	0.924
3rd Day	25	34.8 ± 18.2	25	40.0 ± 23.0	0.230
4Th Day	25	26.4 ± 17.5	25	28.4 ± 19.5	0.581
5th Day	25	22.4 ± 17.8	25	18.0 ± 13.5	0.340

Table 2 shows the mean pain in the first five days and that both groups did not show a significant difference between them (*P*-Value > 0.05) after conducting a paired samples t-test.

### The consumption of analgesic

The same applies to the total number of consumed analgesic pills, as there were no significant differences between the two groups (*P* > 0.05) (Table 3).

**Table 3:** The Descriptive and analytical statistics of the consumption of analgesic pills.

Time of measure	Control Group (N-KT)		Study Group (KT)		T-test
	Count	Mean ± SD	Count	Mean ± SD	P-value
1st Day	25	3.4 ± 1.3	25	3.3 ± 1.3	0.784
2nd Day	25	2.6 ± 1.3	25	2.6 ± 1.6	0.795
3rd Day	25	2.3 ± 1.7	25	2.4 ± 1.9	0.672
4Th Day	25	1.6 ± 1.7	25	1.7 ± 1.7	0.862
5th Day	25	1.2 ± 1.3	25	1.2 ± 1.3	1.000

Table 3 shows the mean number of analgesic pills consumed in the first five days and that both groups did not show a significant difference between them (*P*-value > 0.05) after conducting a paired samples t-test. Therefore, pain levels and analgesic pills consumed were similar between the two groups.

## Discussion

Surgical extraction of third molars is one of the most common surgical procedures in oral and maxillofacial surgery [13]. Recently, surgical extractions of lower third molars have increased due to their increased impaction in the jaws as a result of evolutionary changes [14]. Pain is the most common complications that affect the patient’s quality of life after surgical extraction of the lower third molars. Studies have shown many ways to alleviate these complications. This includes the use of non-steroidal anti-inflammatory drugs and corticosteroids, however, those medications may have toxic and side effects on the body’s systems and organs [15].

Cryotherapy is an easy-to-apply, inexpensive, and repeatable method that is useful in reducing surgical complications through its effect on blood circulation, which causes the narrowing of blood vessels and slow metabolism and cellular exchange [16]. Applying ice packs had an important effect in reducing pain after surgical extraction of the lower third molars. However, its effectiveness in reducing complications remains controversial [17].

Kinesiotape has recently spread as a popular physical method for eliminating complications after surgical extraction of lower third molars after its long-term use in sports medicine and injuries of the musculoskeletal system with the aim to reduce pain and swelling after injuries and to improve the functional performance of the joints [18].

The mechanism of KT depends on the tensile force applied by the adhesive tape after it returns to its original length at the application site. This tension results in convolutions in the skin under the tape, increasing the interstitial distance between the skin and the connective tissues underneath. Which causes reducing in congestion and enhancing in blood circulation and lymphatic drainage in the area. This process has a role in draining fluids and moving them from areas of high pressure to areas of low pressure [19].

In this study, we standardized the operating time to ensure equivalence between the two techniques in terms of difficulty and in terms of postoperative complications such as edema and pain.

In our study, the KT was applied once for 5 days based on several studies [20, 21] that reported the tensile effectiveness of KT is between three and five days, while Patil et al. [7] suggested in their study to replace the KT daily.

To unify the standards between the two treatment methods used in the study, whereby cryotherapy is applied during the first 24 h, KT is applied once, and treatment begins in both groups immediately after the completion of surgical extraction.

Begum and Hossain's study demonstrated that the VAS scale for measuring pain in patients is a subjective and reproducible method to assess pain [12].

The results of the current study showed that the null hypothesis was verified and that there was no statistically significant difference in the level of pain and analgesic consumption between the application of Kinesiotape and cryotherapy when applied after extraction of impacted third molars.

The current study results on day 1; the VAS had approximately equal mean values in the two groups, but the mean number of pills taken was different (3.4 and 3.3, respectively), and on day 5 the VAS had different values in the two groups, but no differences were found concerning the number of pills taken, which may be due to the difference in the pain threshold between the patients, which will require them to consume a different amount of analgesic pills when there is a same level of pain.

In the past few years, interest in using kinesiotape in oral and maxillofacial surgery has increased. Tozzi et al. reported the benefits of KT application in alleviating complications of orthognathic surgery [22]. Benlidayi et al. also reported its effectiveness in alleviating symptoms of TMJ disorders. The role of KT in reducing muscle pain caused by bruxism was reported in Keskinruzgar et al.’s study [23].

The effectiveness of KT has been shown in Ristow et al. studies in alleviating postoperative complications of zygomatic-orbital complex fractures and mandibular fractures. Ristow et al. were among the first to apply KT after surgical extraction of lower third molars [24].

A recent study by Yurttutan et al. reported the role of applying kinesiotape using the Webstrip technique in alleviating complications after surgical extraction of lower third molars [11].

Pain was reduced after surgical extraction of the lower third molars as a result of applying KT by alleviating pressure on pain receptors. This is what was shown in Chiang et al., Tatli et al., De Rocha et al., and Rristow et al. study and Yurttutan et al. study [11, 20, 21, 24, 25].

Also, in the Ertugrul et al. study, which was conducted on patients after knee joint replacement surgery, it was noted that the pain was less in the KT group compared to the group in which cold packs were applied [9].

However, in our study, there were no significant differences in the number of consumed analgesic pills and the amount of pain between the KT group and the control group in which cold packs were applied, this is consistent with the Tozzi et al. study [22].

Genc et al. compared the effectiveness of both KT and a surgical drainage tube in alleviating complications after surgical extraction of impacted lower third molars, according to their study designed with a split-mouth technique. It was found that KT was less effective in reducing edema and pain than a surgical blaster applied at the extraction site, despite the complications. Sepsis may result from the use of the surgical drainage tube [26].

The role of KT in alleviating edema and trismus and improving the patient’s quality of life after surgical extraction of impacted lower third molars was highlighted in the study by Erdil et al. when compared with corticosteroids and NSAIDs, while pain was greater with the use of KT [27].

In the study of de Rocha and her colleagues, KT was applied using the Fan strip technique in the masticatory area of patients after surgical extraction of impacted lower third molars. The results showed that edema and pain were less with the application of KT [21].

Tatli et al.’s study compared the effect of KT and placebo strips in alleviating complications after surgical extraction of impacted lower third molars, and found a positive effect of KT in alleviating pain, edema, and trismus [20].

The study of Tatli [20] was not split-mouth, so the individual effect of each person may have affected the results. For de Rocha, because of there is no placebo and that effect on the results, they only applied KT to one side, but did not apply anything to the other side.

This study had some limitations, such as the inability to apply KT to males who did not want to cut their beard hair at the site of application, and the number of males and females was not equal.

The strengths of this study were that it controlled for many variables that could have distorted the results and that it was the only study to apply the web strip technique to the split mouth.

Within the limitations of the current study, we recommend the use of KT in patients who are receptive to its application, as it reduces the use of pain-relieving medications, and we recommend conducting more studies on its effectiveness when applied in conjunction with cryotherapy.

## Conclusion

Kinesiotape has become one of the physical therapy methods used to alleviate the complications of oral and maxillofacial surgery. The Kinesiotape can be applied in several techniques and we used the web strip technique in this study compared to the application of the cryotherapy. Within the limitation of this study, we concluded that pain and analgesic pills consumed were similar between the two study groups, without one being superior to the other.

## Data Availability

The authors confirm that the data supporting this study’s findings are available in the article; further information can be requested from the corresponding author.
